# Central Serous Chorioretinopathy Classification

**DOI:** 10.3390/ph14010026

**Published:** 2020-12-30

**Authors:** Manuel Vilela, Carolina Mengue

**Affiliations:** 1 Medical School, Federal University of Health Sciences of Porto Alegre (UFCSPA), Porto Alegre 90560-002, Brazil; 2 Institute of Cardiology, University Foundation of Cardiology, Porto Alegre 90040-371, Brazil; carol-mengue@hotmail.com

**Keywords:** central serous chorioretinopathy, central serous retinopathy, macula, retina

## Abstract

Central serous chorioretinopathy is characterized by an idiopathic neurosensory detachment of the retina. This narrative review aims to discuss the classification system used for central serous chorioretinopathy. Based on our current knowledge, there is no universally adopted classification system. This is the result of the unknown aspects related to pathogenesis and clinical spectrum and evolution. The best option could be to aggregate multimodal pieces of information alongside temporal and phenotypic characteristics.

## 1. Introduction

Albrecht von Graefe lived only 42 years but left us with amazing medical contributions. At that time, right after the discovery of the direct ophthalmoscope (1851, Helmholtz), he was the first to report optic neuritis, papilledema, retinal embolism, choroidal tuberculosis, and optic disc glaucoma excavation. He also described the lid lag palpebral sign with regard to goiters, surgical management for acute glaucoma, introduced perimetry, and created a personal technique for cataract extraction [1]. In 1866, he published in the Archiv für Ophthalmologie (this journal is now called Graefe’s Archive for Clinical and Experimental Ophthalmology) a seminal paper entitled “Ueber centrale recidivirende retinitis” that is considered to be the original description of central serous chorioretinopathy (CSC). He noticed similarities with luetic retinitis, but absence of a detectable etiology. The optic disc and retinal vessels had normal aspects and the condition was associated with central suffusion, sometimes with granules inside the macula, being unilateral or bilateral, and causing micropsia with partial reduction in visual acuity. Patients with this condition could have noninflammatory signs during recurrence and prognosis was related to macular function between crises [2].

These are the main basic characteristics that remain unchanged even today. Different names were adopted like “capillarospatic central retinitis,” “angiospatic retinopathy”, “central serous pigment epitheliopathy,” and “idiopathic central serous choroidopathy” until the currently accepted name CSC was consolidated by the original descriptions given by Maumenee [3] (angioscopy) and Gass [4] (fluorescein angiography). The superb contribution made by Gass introduced the concept of choriocapillaris hyperpermeability in the genesis of CSC (50 years ago, based only on fluorescein observations). Thirty years later, his theory was certified by indocyanine green angiography observations [5,6].

There are more than 2400 papers containing the term CSC held on the MEDLINE/PubMed Database between 1948 and August 2020 [7]. However, thus far its etiology, pathogenesis, and management remain unclear. The incidence of CSC is around 10 cases per 100,000 men and 1.7 per 100,000 women. Different risk factors like corticosteroids use, anxiety trait, pregnancy, and endogenous hypercortisolism have been described [8,9,10].

There is no universally accepted classification for this disease, and this is a crucial problem. Different proposals consider factors like duration of subretinal fluid, the spectrum of phenotypes, existence of a causative factor, a grade of activity, and recurrences. However, a consensus is still lacking, and this is of the utmost importance for correctly analyzing and defining best case management in a standardized and prospective manner [8,9,10].

The aim of this review was to examine the current classification status of CSC and aggregate complementary information on this subject.

## 2. Classification Models

CSC is one of the most frequent retinal situations in ophthalmological practice, however, it still has very many unexplained points. There is some knowledge about demographic behavior, such as predominance of the male sex and ages ranging from 39 to 51 years (although it can occur later in life, sometimes mimicking age-related macular degeneration, and in women with a particular tendency of accentuated damage). Ethnic variations seem to show that some populations could have more incident and/or severe clinical situations (multifocal and bilateral), besides higher pachychoroid prevalence [8,9,10,11,12,13,14,15].

Its natural history is based on observations of small samples, with different follow-up periods, and measurements performed using unequal parameters (fluorescein angiography, visual acuity, fundus posterior biomicroscopy, and optical coherence tomography). Many of these studies analyzing the spontaneous course of this condition were conducted centered on the presence or absence of symptoms or fluorescein leak and were conducted years before the introduction of optical coherence tomography (OCT) [9,10,14,15]. Mrejen et al. [16] recently reported results of a 10 year study of 217 eyes with CSC, and, using multimodal images, they found that 55% kept visual acuity VA of 20/40 or more, but 12.8% became legally blind in both eyes. Impairment of the ellipsoid zone (41.5%) and external limiting membrane (67.3%) shown by OCT are directly associated with final VA. Furthermore, subretinal neovascularization (type 1) was detected in 24%, cystoid degeneration in 21.7%, and subretinal fibrosis in 11.5%. Ersoz et al. [17] retrospectively analyzed 811 patients with CSC in order to describe demographics, risk factors, and morphological aspects. They found bilateral disease in 42% (64.2% of chronic cases had bilateral disease) and pigmented epithelial detachment in 80.7%, while subfoveal choroidal thickness was greater in multifocal cases.

The 4–6-month criteria for observing acute forms are based on damage seen in the photoreceptor layer and other neuroretinal atrophy aspects [18,19,20,21]. However, functional impairment (visual acuity and contrast sensitivity) and outer nuclear layer thickness reduction in shorter periods (<3 months) have been reported [22,23,24,25]. Behnia et al. [23] showed that in acute CSC with duration of less than 1 month sequels in contrast sensitivity were significantly worse in control group patients. Hata et al. [24] measured outer nuclear layer (ONL) thickness in patients with different symptom duration lengths and showed that even those with only 1-month duration had significant ONL thinning and progressive lesion in the presence of persistent subretinal fluid. When using adaptive optics scanning laser ophthalmoscopy (AO SLO) in an observational case series to compare pathological changes in photoreceptors in normal eyes versus eyes with resolved central serous chorioretinopathy, Ooto et al. [26] found abnormal cone mosaic pattern and lower cone densities in CSC eyes. These modifications were associated with functional visual loss. Through SD-OCT analysis, Hasegawa et al. [27] showed that in patients with acute CSC, resolution time was associated with visual disturbances and modification in central foveal thickness and photoreceptor outer segment length. Ross et al. [28] conducted an experimental study, which demonstrated that acute subretinal fluid causes a significant loss of rods, reduction in ONL thickness, and association with release of a high-mobility group box 1 protein (HMGB1).

This unsolved controversy compromises temporal classification. Observations with regard to external retina and choroid in OCT may help us to define the best moment to adopt treatment. In a retrospective analysis, Ambiya et al. [29] showed that thinner choroid may indicate progression to chronic stages and will need treatment. Why did some patients with a variable number of leaks lasting more than 4 months not show retinal pigment epithelium (RPE) changes? In addition, why do patients with less than 3–4 months of symptoms have permanent damage? Yu et al. [30] reported a 10-fold difference in symptom duration according to the specific patterns of damage to the photoreceptor layer found by using OCT. Thus far, it has not been possible to define precisely which patients with initial or late manifestations of CSC will recover spontaneously [31].

Even so, temporal and phenotype criteria (structural changes) are currently the only universally accepted criteria for classifying and distinguishing CSC. However, overlapping discrepancies and terminologies are excessive even among experts, and this creates a strong limitation in the development of management strategies [32,33]. Singh et al. [33] conducted a multicenter survey with multimodal images of 100 acute and chronic CSC cases. Agreement between classification and descriptor terms used among the graders was poor. The consistent diagnosis of CSC was significantly higher than in chronic or recurrent cases. Another unsolved question is related to the spectrum and variability of this disease. CSC has a wide spectrum ranging from asymptomatic cases to a diffuse and severe condition. Sartini et al. [34] conducted a systematic review, which described different aspects of a very rare variant of the CSC spectrum named “Bullous” CSC and emphasized the value of early recognition and the challenges of differential diagnosis.

Is CSC a unique and continuous situation that evolves from an acute phase or is it composed of distinct situations? An unknown number of patients could have an extramacular disease or be unaware of the disease. This can contribute to the high proportion (between 8% and 73%) of advanced cases, which did not have a previous “acute” episode [9,35].

The acute form is defined by the presence of sensorial macular detachment with one or several leaks and RPE alterations related to small pigment detachments (PED) with recovery within 3–6 months with no expressive functional sequels. The chronic form (also called “diffuse retinal epitheliopathy”) is characterized by the presence of damage at the RPE level (bumps, atrophy, descending or gravitational tracks, possible presence of flat RPE detachments, multiple leak points, cystoid macular degeneration, subretinal fibrosis, and macular neovascularization). Persistent or “nonresolving” CSC is an acute situation with longer SRD duration (4–6 months, considering the estimated date of initial symptoms) and possibly showing photoreceptor changes in OCT. Recurrent CSC is defined by a new acute episode after complete previous serous detachment resolution. Inactive CSC refers to patients with previous episodes but without current serous detachment [9].

This proposal does not embrace all scenarios and creates semantic overlapping. Basically, an acute disease occurs rapidly and usually has limited duration. The chronic situation means something that lasts a long time, in general, many months. Expectation of cure is also used for this distinction, and some chronic diseases require long-term management. Sometimes an acute process could result in chronic one, and chronic diseases present periods of acute recurrences. Some diseases will progress from an acute to a chronic phase, and some chronic process can stay silent or latent for years before an acute event appears. The recurrence of the same disease in the same organ(s) after complete recovery and sometimes with residual scars is considered, most of the time, in general medicine, as a chronic unsolved process [36,37,38].

Therefore, it is not easy to correctly classify CSC, and the confusion and discrepancies could be the result of the use of specific timeframes. Should patients who have acute symptoms in one eye but asymptomatic RPE signs in the contralateral eye (or even previously unnoticed other focal areas of RPE changes in the diseased eye) be considered as having an acute or a chronic situation? In a prospective case series, Gupta et al. [39] reported that patients with idiopathic CSC showed RPE bumps (94%) and RPE detachments (11.8%) in unaffected asymptomatic eyes. Additionally, what about those patients who develop an acute disease in the second eye (initially normal) after a unique inactive but symptomatic episode in the first eye? Simultaneous CSC is reported as ranging from 4% to 42% [17,40]. CSC recurrence is very common, about 15–50% will have a second episode and around 30% of them will have a further episode [8,10]. Is persistence that seems like a prolonged convalescence period with the same or a new leak point not an evident trend of a chronic illness?

With regard to chronic CSC phenotypes, it is of the utmost importance to define which are the earliest clinical signs, anticipating the easy but dramatic recognition of extensive damage. Should focal RPE mottling, atrophy, sometimes with atrophic halo developed by the pre-existence of fluid in the symptomatic or asymptomatic eye be considered to be a positive clue?

Another confounding aspect is related to misdiagnosis. A lot of conditions can mimic CSC, including choroidal diseases (neovascularization and hemangioma), inflammatory situations (Vogt-Koyanagi-Harada, posterior scleritis, and posterior uveitis), retinal vascular disease (incipient retinal angiomatosis proliferation and idiopathic juxtafoveolar retinal telangiectasis), anatomic abnormalities (optic disc pit and dome-shaped macula), and others, such as uveal effusion syndrome [41].

## 3. Complementary Analysis

Continuous multimodal studying of each case is essential and one useful aspect is the identification of biomarkers of high-risk cases. Some of these signs are included in the Table 1. We can aggregate the nature of the episode as an alternative and complementary way to classify the CSC spectrum. With effect from first recognition and during long-term follow-up, controlling biomarkers could help to define the case management recommendation [42,43,44,45,46,47,48].

**Figure 1:**
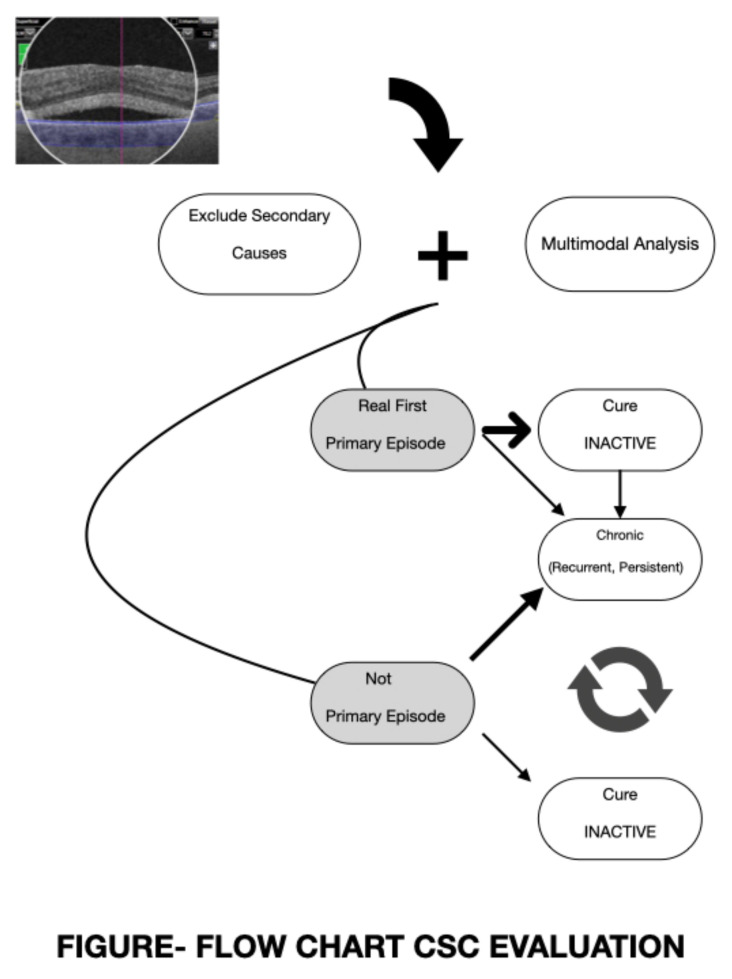
Basic steps: (1) define if your patient has a real first one episode or not and (2) a first episode can go to complete resolution (long-term follow-up) or become recurrent or persistent (chronic disease). In “not primary episode,” three options are possible: (1) become inactive, (2) evolve like a refractory disease, or (3) evolve in a cyclical way.

The first step in each CSC case is to define whether or not we are dealing with a real first episode. This complementary analysis should begin by excluding secondary causes (corticosteroids, MEK-Inhibitors, Cushing syndrome, and other frequent causes of misdiagnosis). Following this, a detailed and regular study of the information offered by multimodal resources in both eyes is mandatory. Previous asymptomatic signs must be investigated in both eyes. Multimodal studies can identify different possibilities. It could be a real first lifetime acute episode or a recurrent episode that has gone unnoticed in the diseased eye or the contralateral eye. Once we have detected a patient with sequels from prior undetected disease, immediate therapy probably becomes a strong necessity (Figure 1).

Older patients are subject to greater risks, such as macular neovessels or chronic repercussions associated with this situation [8,10,27]. Detection of RPE focal changes in any retinal area (mottling, atrophy, bumps, gravitational, or descending tracks) [8,12,14,21,29] by posterior fundus biomicroscopy or any other resource is a strong indicator of a recurrent situation. Detection of multiple pinpoint leaking and/or new single focal leaking identified during intravenous fluorescein angiography (IVFA) are associated with longer evolution [14,21].

Autofluorescence and infrared autofluorescence evidence, such as persistence of hyperautofluorescence of the serous detachment zone, a granular pattern sometimes associated with the appearance of descending tracks, could be associated with long evolution or a recurrent situation [42,43,44,45,46,47,48,49,50,51,52,53]. IVFA in acute CSC typically shows two patterns: inkblot or smoke stack. These points are often close to a pigment epithelium detachment. It is important to remember that patients with near-normal intravenous fluorescein angiography (IVFA) may have subretinal fluid. Chronic CSC may show the RPE defects and multiple sites of leakage. Indocyanine green angiography (ICG) is not a widely available resource but can identify higher risks in cases with multiple leak zones without IVFA correspondence [5,6,17,35].

OCT has given us many contributions to this critical analysis. One of the most relevant aspects has been detection of pachychoroid. Initially, the term was used to describe an increase in choroidal thickness (CT), although, nowadays, the morphologic changes observed in the choroid are essential for diagnosis. These include dilated vessels in Haller’s layer, thinning of Sattler’s layer, and choriocapillaris, with or without RPE changes over these areas [54,55]. There is no consensus on CT threshold limits because there are a lot of variables that can influence those measurements. Moreover, CSC can occur with or without pachychoroid, while thickness of 500 micron or more is related to chronicity [25,56,57]. The spectrum of pachychoroid disease include CSC, pachychoroid pigment epitheliopathy, pachychoroid neovasculopathy (can progress to polypoidal lesion), focal choroidal excavation, and peripapillary pachychoroid syndrome. Detachment of retinal pigment epithelium (PED) higher than 50 micron or associated with the presence of the double sign that indicates irregular and flat vascular PED, a large amount or recurrent subretinal fluid, hyperreflective choroidal vessel walls, intra- and subretinal hyperreflective dots materials, external limiting membrane discontinuation, thinning of the outer nuclear layer, elongation of the photoreceptor outer segments, retinal cystoid degeneration, pigment deposition, fibrinoid reaction, choroidal rifts, and reduction in the ganglion cells complex are all indicative of severe forms with different functional prognosis [9,10,11,14,25,56,57,58,59,60,61,62,63,64] (Table 1).

Use of OCTA has expanded facility in detecting nonexudative macular neovascularization or mimicking situations. The prevalence range of macular neovascularization diagnosis using OCTA in chronic CSC is 8.3–44.8% [65,66,67,68,69,70,71]. These results provided by OCTA are much better than those from isolated IVFA. They are more specific than the combination of IVFA/OCT and are quite similar to ICGA [8,9,10,11,12,13,14]. Other relevant signs in OCTA are flow void zones and darker areas. These zones mean that microcirculatory deficiencies are detected in the diseased and fellow eyes [72,73].

Reduced implicit time in multifocal electroretinogram has been described as an indicator of compromised functional prognosis [74]. Otherwise, reduced retinal sensitivity is detected through microperimetry in areas of RPE irregularities or junctional disruption and persistent subretinal fluid [75,76,77].

## 4. Conclusions

In conclusion, thus far, there is no ideal classification. Building one universally accepted kind of classification is mandatory to help us to avoid overlappings and organize the management of these cases. Meanwhile, the use of complementary signs should be employed along with temporal and phenotype observations to help to improve our knowledge and management decisions.

## Figures and Tables

**Table 1:** Critical signs of central serous chorioretinopathy (CSC) in multimodal images.

Multimodal Images	Critical Signs
**Retinography**	RPE focal changes (mottling, atrophy, bumps, tracks) [8,12,14,21,32]
**Fluorescein angiography**	A new leak point in a different location [14]
	Multifocal leak pinpoints [14,21]
**Autofluorescence**	Descending or gravitational tracks progression [49,50,51,52]
	Persistent hyperautofluorescence in the zone of serous detachment [41,42,43,44]
	Increase granularity after resolution [42,43,44,45]
**Infrared autofluorescence**	Granular pattern [53]
**ICG**	Multifocal leak zones without correspondence in IVFA [5,6,17,35]
**OCT**	CT > 500 micron [25]
	PED height > 50 micron [59]
	Large amount or recurrent subretinal fluid [9,14]
	Choroidal hyperreflective dots [9,10,14]
	Hyperreflective choroidal vessel walls [9,10,14]
	Intra- and subretinal hyperreflective dots and material [60]
	Thinning and atrophy of the outer nuclear layer [61]
	External limiting membrane discontinuity [62]
	Cystoid degeneration [11]
	Elongation of the photoreceptor outer segments [9,10,32]
	Double sign at external retina (irregular, flat, and dense RPE detachment) [65]
	Fibrinoid reaction [9,10]
	Pigment deposition [9,10,32]
	Choroidal rift [58]
	Reduced ganglion cell complex thickness [64]
**OCTA**	Subretinal neovascularization [65,66,67,68,69,70,71]
	Flow void zones [72,73]
**MfERG**	Reduced implicit time [74]
**MICROPERIMETRY**	Reduced retinal sensitivity in areas of RPE irregularities or IS/OS disruption [75,76]
